# Cutaneous metastases of breast carcinoma: a case report

**DOI:** 10.1186/1757-1626-2-71

**Published:** 2009-01-21

**Authors:** Sergio Vano-Galvan, Paula Moreno-Martin, Irene Salguero, Pedro Jaen

**Affiliations:** 1 Dermatology Service, Ramony Cajal Hospital, University of Alcala, Madrid, Spain; 2 La Princesa University Hospital, Madrid, Spain

## Abstract

**Background:**

Cutaneous metastases can have variable clinical appearances and can mimic benign skin lesions. They are usually seen in patients with advanced disease, but they can be the presenting lesion.

**Case presentation:**

A 48-year-old woman presented with a 3-month history of progressive appearance of multiple asymptomatic nodular lesions on the chest. The recognition of cutaneous metastases often dramatically alters therapeutic plans, especially when metastases signify persistence of cancer originally thought to be cured. The most common tumor to metastasize to the skin is breast cancer.

**Conclusion:**

Every practitioner should be highly suspicious of acute-onset, persistent, firm papulonodules, especially when they develop on the chest.

## Case presentation

A 48-year-old woman presented with a 3-month history of progressive appearance of multiple asymptomatic nodular lesions on the chest. One year earlier she was diagnosed of invasive ductal adenocarcinoma of the left breast and received treatment with neoadjuvant chemotherapy followed by a left modified radical mastectomy and axillary lymph node dissection.

The patient referred an intense asthenia since a few months ago but not local symptoms such as pruritus or pain. The remainder of her medical history was non-contributory. She had no recent additions to or changes in her medications.

**Figure 1:**
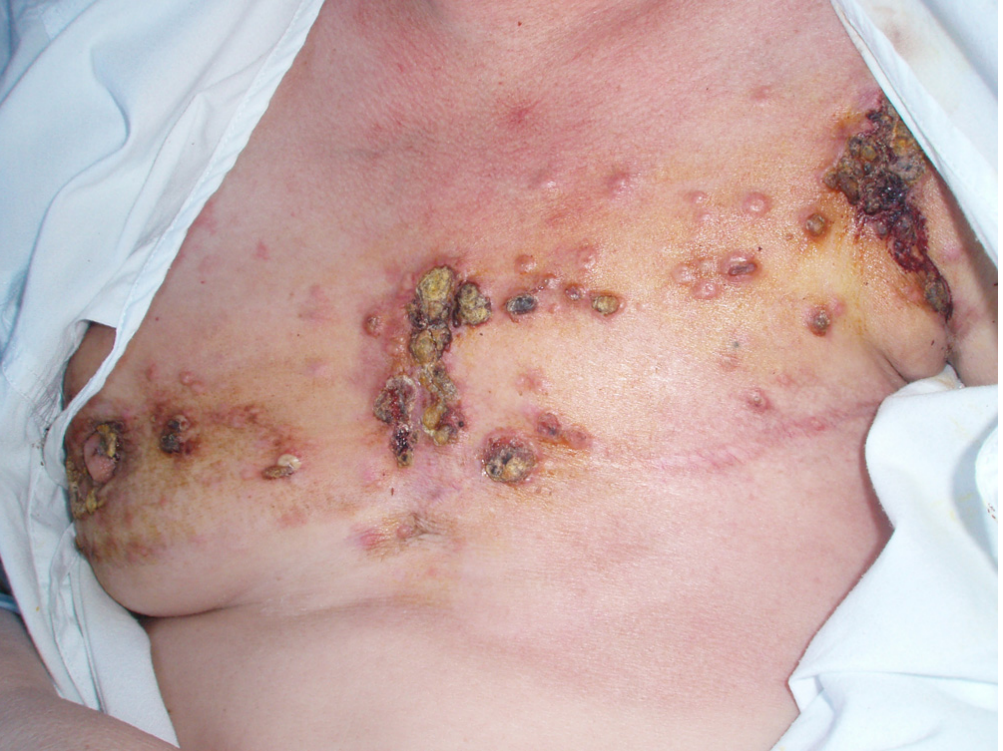
**Multiple round-oval, firm, flesh colored exophytic nodules and exophytic lesions, located on the chest**.

Physical examination revealed multiple round-oval, flesh colored exophytic nodules and exophytic lesions, with very firm consistency on palpation, scattered on the chest, some of them ulcerated (Figure 1). Lesions affected both right and left breast, and were non-painful. The skin around nodules was firm and infiltrated on palpation.

The rest of cutaneous as well as physical examination was unremarkably. Chest X-ray and TC-study showed multiple nodular lesions involving both lungs. A skin biopsy was performed and it was confirmed the diagnosis of cutaneous metastases (CM) of breast carcinoma.

## Discussion

CM can have variable clinical appearances and can mimic benign skin lesions. They are usually seen in patients with advanced disease, but they can be the presenting lesion [1]. The recognition of CM often dramatically alters therapeutic plans, especially when metastases signify persistence of cancer originally thought to be cured.

The overall incidence of CM is 5.3%. The most common tumor to metastasize to the skin is breast cancer. The metastatic capabilities of breast carcinoma are well described. Major sites of extramammary involvement, in decreasing prevalence, are the lungs, bones, liver, adrenal, brain, skin and kidneys. The chest is the most common site of CM. Every practitioner should be highly suspicious of acute-onset, persistent, firm papulonodules, especially when they develop on the chest [2].

Breast cancer is the most commonly diagnosed cancer in women and the second leading cause of cancer deaths among women in the United States. Many women diagnosed with breast cancer will achieve a cure with surgery followed by adjuvant chemotherapy, hormonal therapy, or radiation therapy; however, some breast cancer survivors will develop locally recurrent disease. About 20% of women with a history of early breast cancer will ultimately develop metastases [3]. CM are one of the most distressing presentations of locally recurrent breast cancer [4].

CM in breast carcinoma have several morphologic characteristics. Localized nodules represent the most common presentation, occurring in 10% of patients [5]. Cicatricial morphology is another pattern and is characterized by asymptomatic plaques often appearing on the scalp and associated with hair loss [5]. Fibrotic-type cutaneous metastatic spread to the trunk has been described as an "encasement of armor" or en cuirasse, given the development of a hard, leathery plaque. Bullous lesions are another type of presentation of cutaneous metastatic breast cancer, but their appearance is uncommon [5]. Finally, CM may present in the context of generalized inflammation of the affected area and typically presents with erythema and edema resembling cellulitis.

A biopsy of the skin helps in confirming a diagnosis of tumor. The pattern noted and the microscopic appearance often suggests the likely tissue of origin. The initial diagnosis can be made by examining frozen sections, but the final diagnosis should be reserved until permanent sections are included. Generally, the histologic features of the metastases are similar to the primary tumor, although metastases may be more anaplastic and exhibit less differentiation.

Effective treatment depends on treatment of the underlying tumor. Palliative care is given if lesions are asymptomatic and the primary cancer is untreatable. This care includes keeping lesions clean and dry and debriding the lesions if they are bleeding or crusted. Hydrocolloid dressings may be used to help prevent secondary infection.

## Competing interests

The authors declare that they have no competing interests.

## Authors' contributions

SV-G and PM-M wrote the initial draft of and helped revise the manuscript. IS obtained consent from the patients and helped revise the manuscript. PJ assisted with manuscript revision. All authors read and approved the final manuscript.

## Consent

Written informed consent was obtained from the patient for publication of this case report and accompanying images. A copy of the written consent is available for review by the Editor-in-Chief of this journal.

## References

[B1] Sariya D Ruth K Adams-McDonnell R Cusack C Xu X Elenitsas R Seykora J Pasha T Zhang P Baldassano M Lessin SR Wu H Clinicopathologic correlation of cutaneous metastases: experience from a cancer center Arch Dermatol 2007 143 613 620 10.1001/archderm.143.5.613 17515511

[B2] Krathen RA Orengo IF Rosen T Cutaneous metastasis: a meta-analysis of data South Med J 2003 96 164 167 10.1097/01.SMJ.0000053676.73249.E5 12630642

[B3] Stevanovic A Lee P Wilcken N Metastatic breast cancer Aust Fam Physician 2006 35 309 312 16680209

[B4] Moore S Cutaneous metastatic breast cancer Clin J Oncol Nurs 2002 6 255 260 10.1188/02.CJON.255-260 12240484

[B5] Lookingbill DP Spangler N Helm KF Cutaneous metastases in patients with metastatic carcinoma: a retrospective study of 4020 patients J Am Acad Dermatol 1993 29 228 236 10.1016/0190-9622(93)70173-Q 8335743

